# Rapidly Progressive Glomerulonephritis: A COVID-19 Case Report

**DOI:** 10.7759/cureus.37767

**Published:** 2023-04-18

**Authors:** Ali Tahir, Jasmit Walia, Timothy Daly, Alexandra Gradzka, Ruslan Banai

**Affiliations:** 1 Internal Medicine, St. Luke's University Health Network, Bethlehem, USA

**Keywords:** pericarditis, rapidly progressive renal failure, renal failure, pauci-immune glomerulonephritis (gn), covid 19, antineutrophil cytoplasmic antibody (anca) associated vasculitis (aav)

## Abstract

Anti-neutrophil cytoplasmic antibody (ANCA) associated vasculitis is a systemic autoimmune disease that typically presents as a multi-organ manifesting disease of unclear etiology that can predispose to rapidly progressive glomerulonephritis (RPGN). If left untreated, ANCA-associated vasculitis can be fatal, and RPGN can progress to irreversible renal failure. Environmental and genetic factors have been implicated in the pathogenesis of this vasculitis. Coronavirus disease (COVID-19) has been noted to have various physiologic impacts on the body, with literature indicating possible autoimmune effects. We present a rare case of ANCA-associated vasculitis in an elderly male with no known autoimmune history after a recent illness with COVID-19. The patient had been seen as an outpatient with progressively declining renal function until he presented to the hospital with acute renal failure and pericarditis. Workup revealed elevated anti-myeloperoxidase antibody (MPO-AB) and perinuclear ANCA (p-ANCA) antibodies with a biopsy confirming focal cresenteric glomerulonephritis, and the patient was initiated on steroid therapy with notable improvement and a return to baseline kidney function.

## Introduction

Rapidly progressive glomerulonephritis (RPGN) is a rare form of vasculitis, with the pauci-immune type being the most common form of crescentic glomerulonephritis at around 65%-70%. The majority of these cases are found to be positive for anti-neutrophil cytoplasmic antibody (ANCA) [1]. ANCA-associated vasculitis (AAV) is a small- and medium-sized vessel disease typically with multi-organ involvement, including pulmonary, renal, dermatologic, and neurologic manifestations. However, they may also present with non-specific systemic symptoms such as fever, fatigue, and myalgias. Rarely, it may only involve the kidneys, leading to the classification of “renal-limited vasculitis” [2].

It is unclear as to what the cause of AAV is; however, studies have pointed toward both genetic and environmental components [3,4]. A wide range of complications has been reported since the rise in coronavirus disease (COVID-19), which have been predominantly respiratory but also include and are not limited to hematologic, cardiac, dermatologic, and renal involvement [5]. Acute kidney injury (AKI) has been a common complication of COVID-19 with varying incidence rates based on different studies, with recorded rates as high as 56.9% and variable rates of renal replacement therapy (RRT) depending on AKI severity [6]. However, reports of vasculitis associated with COVID-19 have been low, with even fewer reports of perinuclear anti-neutrophil cytoplasmic antibody (P-ANCA)-associated vasculitis.

## Case presentation

An 82-year-old male with a past medical history of hypertension, gastroesophageal reflux disease, and hyperlipidemia initially presented to the hospital with gradually worsening pleuritic chest pain for 3-4 weeks before admission. In addition, the patient noted having worsening urinary incontinence. During the initial work-up, he was found to have pericardial effusion on a transthoracic echocardiogram and concomitant acute renal failure with a creatinine of 4.03 mg/dL (ref: 0.60-1.3 mg/dL).

The patient was treated as an outpatient before admission for what was assumed to be arthritis-related bilateral hand pain, swelling, and stiffness. Lab work revealed negative anti-nuclear antibody (ANA), rheumatoid factor (RF), Sjogren’s, and anti-cyclic citrullinated peptide (anti-CCP) biomarkers with elevated C-reactive protein (CRP) of 24.9 mg/L (ref: <3 mg/L) and erythrocyte sedimentation rate (ESR) of 81 mm/hour (ref: 0-19 mm/hour). The patient was treated with oral steroid therapy, which led to symptomatic improvement. Notably, he was found to be COVID-19 positive four weeks after that (eight weeks before admission) and was treated with Nirmatrelvir and Ritonavir as an outpatient with no hospitalization required. The patient was fully vaccinated, with one booster given five months before infection. During the workup, he was noted to have a baseline creatinine of 1.1-1.3 mg/dL (ref: 0.60-1.3 mg/dL) that had been trending upwards on outpatient lab work and had risen to 4.03 mg/dL by the time of admission. Initial workup also revealed worsening anemia with a hemoglobin of 8.9 g/dL (ref: 12.0-17.0 g/dL), rising blood urea nitrogen (BUN) at 54 mg/dL (ref: 5-25 mg/dL), and a urinalysis revealing microscopic hematuria with proteinuria.

Given hemodynamic stability, the patient’s pericardial effusion was medically managed, and he was started on colchicine, given concerns for pericarditis. 

The patient had been taken off all nephrotoxic agents and received intravenous fluid hydration with no improvement in creatinine function. Renal ultrasound was unremarkable without evidence of hydronephrosis.

**Figure 1:**
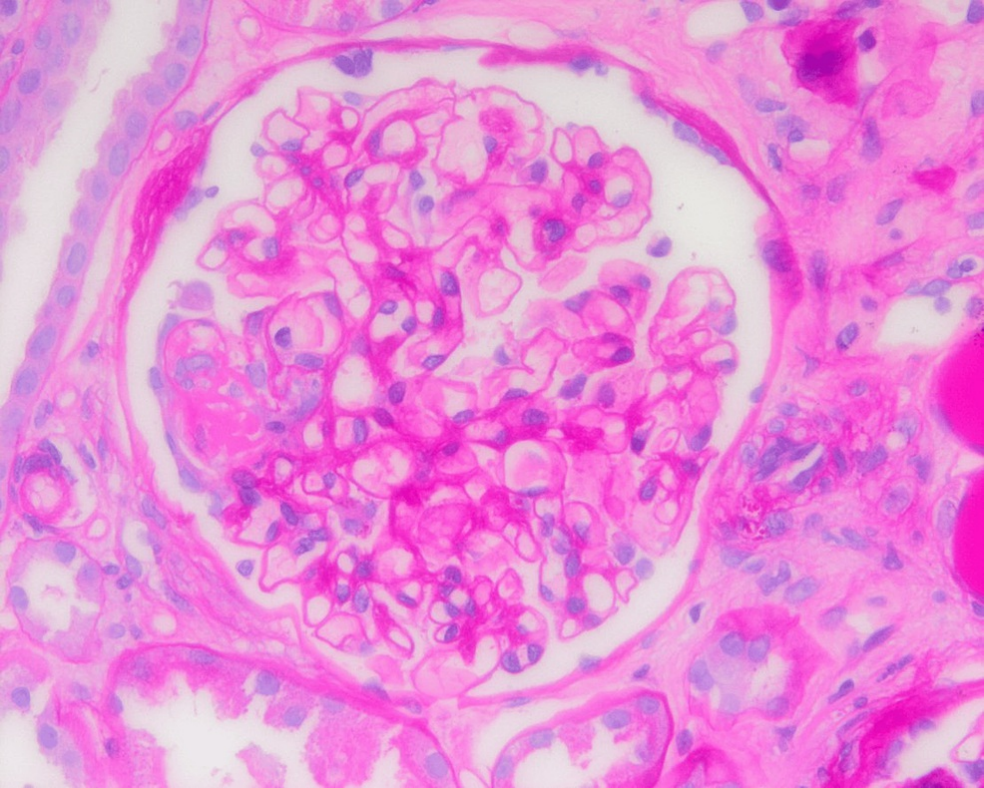
Single glomerulus with foci of fibrinoid necrosis.

**Figure 2:**
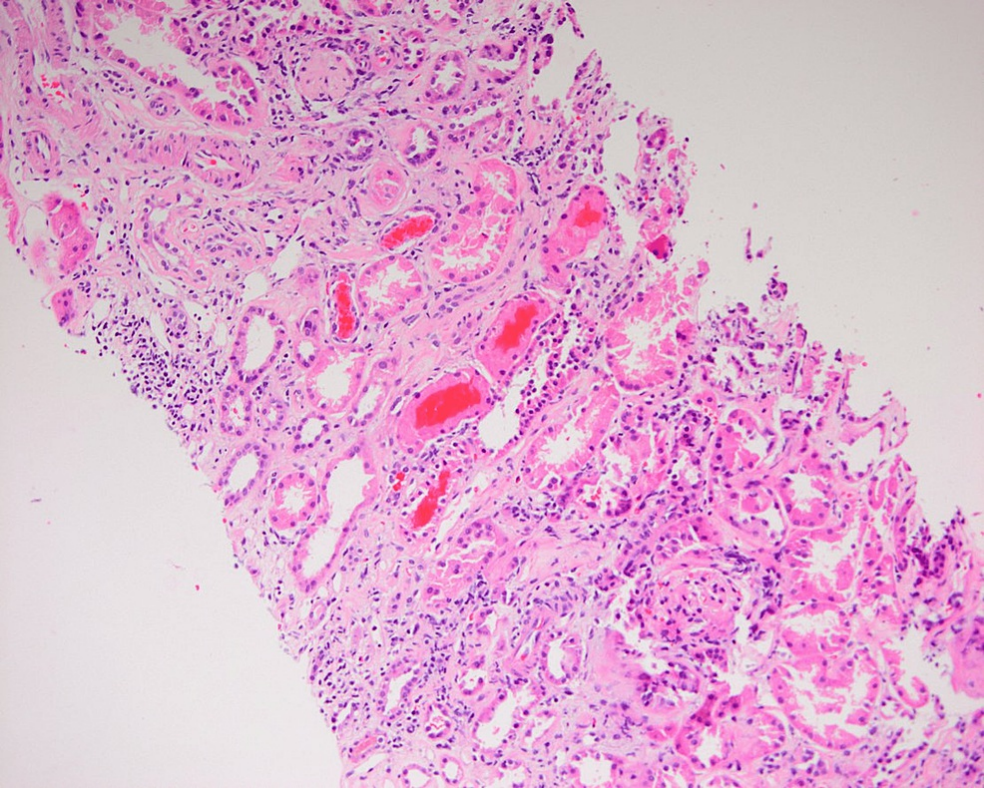
Core biopsy revealed some intimal fibrosis with signs of tubulointerstitial inflammation and collections of inflammatory cells. Hypercellularity is noted throughout, and moderate interstitial fibrosis and tubular atrophy can be appreciated.

**Figure 3:**
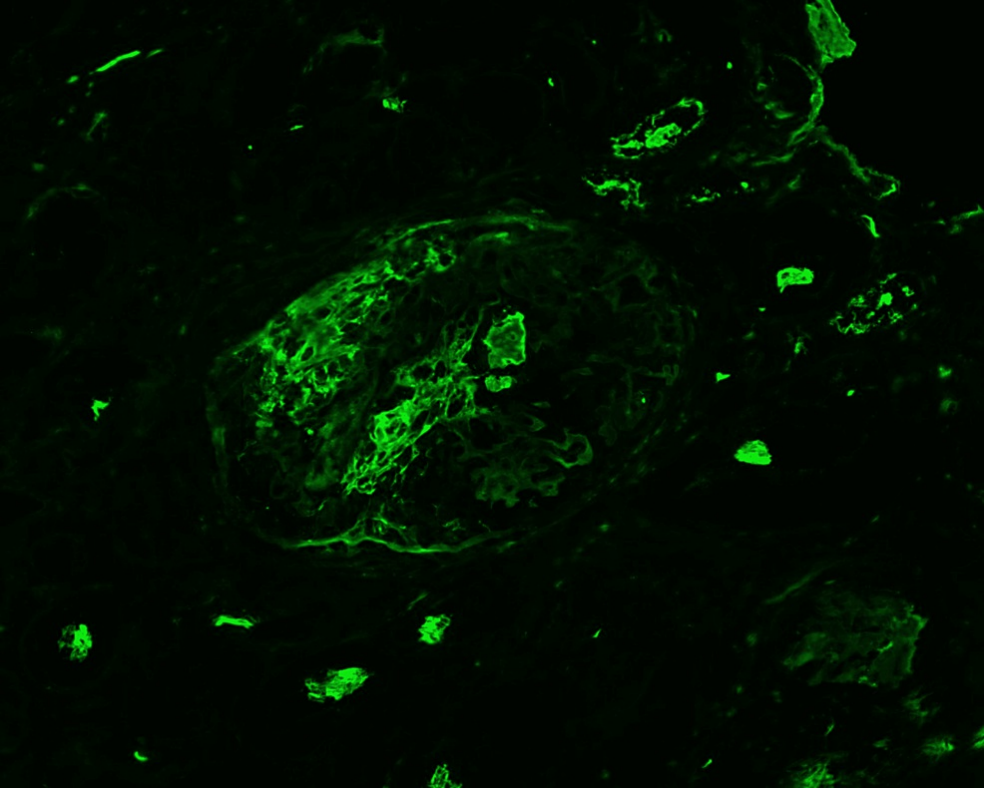
Immunofluorescent staining shows a glomerulus with crescentic fibrin deposition. A segmental glomerular reaction for fibrinogen within Bowman's space, representing areas of necrosis and crescent formation, is present. No significant reaction for IgG, IgA, IgM, C3, C1q, kappa, or lambda was noted.

**Figure 4:**
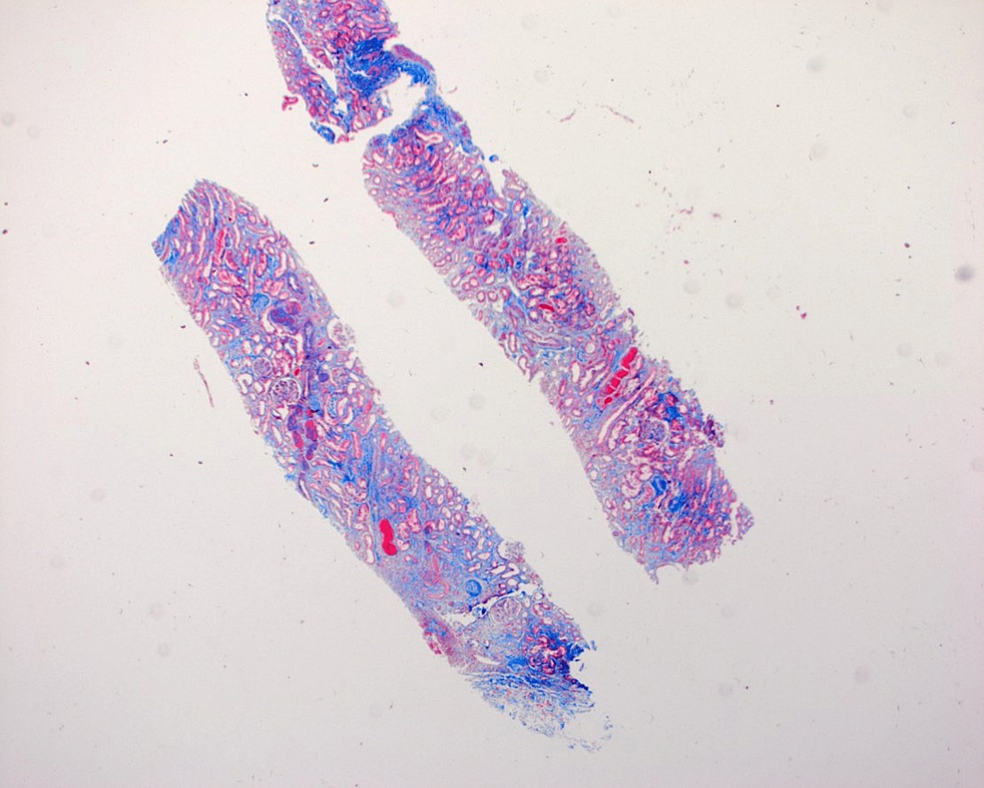
Core biopsy with trichrome staining on low power demonstrating dense collagenous deposition.

**Figure 5:**
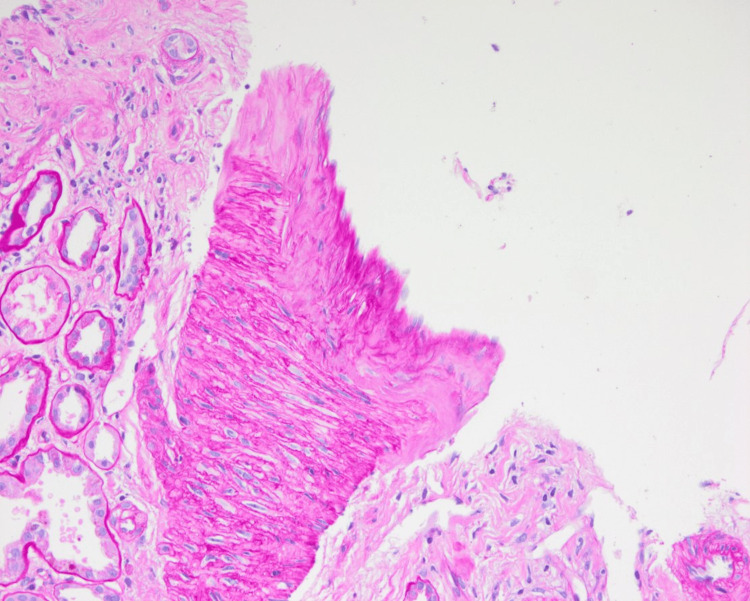
Vessel seen showing mild arterial intimal fibrosis and no significant arteriolar hyalinosis or arteritis.

The patient underwent an autoimmune workup. Negative findings include negative ANA, anti-double-stranded DNA antibodies (anti-dsDNA), and C3 and C4 complements. The repeat CRP was 129 mg/L with an ESR of 100 mm/hour. The urine/protein creatinine ratio was 1.5 (ref: 0.00-0.10). He was found to have anti-myeloperoxidase antibody (MPO-AB) levels of >100.0 U/mL (ref: 0.0-9.0 U/mL) and a P-ANCA titer of 1:640 (Neg: <1:20 titer). A renal biopsy was obtained with a diagnosis of focal crescentic glomerulonephritis, pauci-immune type, with the findings noted in Figures 1-5. Of the 24 glomeruli seen, seven demonstrated global glomerulosclerosis. Moderate interstitial fibrosis and tubular atrophy were present in 30%-40% of the cortex. 

After a biopsy was taken, the patient was started on pulse-dose intravenous methylprednisolone sodium succinate 250mg twice a day therapy for three days while inpatient, with subsequent lab work showing improvement in his kidney function. Upon completion of intravenous therapy, the patient was placed on oral Prednisone 60mg, followed by a taper lasting seven weeks. He was started on Rituximab treatment at 1g every two weeks for two doses as an outpatient. The patient did not require dialysis during the hospitalization. A repeat echocardiogram after discharge showed improvement in pericardial effusion. Upon follow-up, the patient was found to have continued elevated titers and received a second cycle of Rituximab at the same dose with subsequent improvement. The patient continues to follow up for outpatient monitoring.

## Discussion

The association between COVID-19 and vasculitis has been uncommon, and the association between COVID-19 and AAV is even less so. Our patient presented eight weeks after testing positive for COVID-19 with mild symptoms. Before hospitalization, the patient had unexplained worsening kidney function and was found to have pericardial effusion and acute renal failure (ARF) on admission. While the ARF was found to be secondary to AAV, the etiology of the pericarditis remained unclear, with a differential diagnosis of uremic pericarditis in the setting of ARF versus viral pericarditis in the setting of recent COVID-19.

Since the initial manifestation of COVID-19, there has been extensive research and review on the clinical manifestations and long-standing effects of the disease process [5,7]. While the severity of the disease has decreased with the introduction of vaccines, there remains the question of the manifestations of illness and the prevalence of “long COVID” [7,8]. However, definitions of what qualifies as long-term COVID remain inconsistent and have predominantly focused on unexplained symptoms rather than organ dysfunction manifestations, such as pericardial effusion or renal injury [7].

Since the COVID-19 pandemic, there have been few incidences of AAV alongside recent COVID-19 infections in the adult population without known underlying immune disorders. Symptom manifestations varied widely in each case, including pulmonary and dermatologic complications, in addition to the findings of glomerulonephritis. In all of these cases, patients were found to have pauci-immune glomerulonephritis with either cytoplasmic ANCA (C-ANCA) or P-ANCA findings [9-12]. Given the incidence of AKI in COVID-19, it can be difficult to ascertain the primary etiology of kidney injury. However, in the case of our patient, he continued to have worsening kidney function weeks past his original COVID-19 diagnosis.

The question that arises is what impact COVID-19 has on autoimmune or vascular physiology. It does appear that the disease has properties similar to those seen in autoimmune diseases, and in the case of AAV, specifically the neutrophil extracellular trap production (NET), which has been shown to be increased in COVID-19. It is not unreasonable that there is likely some association between AAV and COVID-19 in this presentation. It is possible that either AAV was a byproduct of COVID-19 or that COVID-19 unmasked an underlying genetic etiology [2,13-15].

In the setting of our patient, the presence of pericardial effusion may have been due to uremic pericarditis in the setting of a worsening kidney injury but may also be related to the immune insult suspected to be instigated by COVID-19, leading to both the pericardial effusion and renal failure. There have been rare instances of COVID-19 presenting with pericardial effusion, which may lend itself to supporting the immune insult that the disease process may affect [16,17].

Also taken into consideration was the antiviral therapy that the patient received. As far as is documented in the literature, there are no documented consistent renal adverse effects to Nirmatrelvir and Ritonavir.

## Conclusions

COVID-19 has presented with a wide range of complications, and its pathogenicity continues to be studied. Given the unclear etiology of AAV in general, it remains uncertain whether COVID-19 had induced AAV in our patient or had unmasked an underlying autoimmune process. In this case, we presented an otherwise healthy individual with new-onset renal failure from a mild COVID-19 infection. In the setting of a recent diagnosis, it is important to keep vasculitis on the differential for a patient with worsening renal function, especially with the decrease in severe COVID-19 cases in the setting of vaccinated patients.
